# Detection of freezing of gait in Parkinson's disease from foot-pressure sensing insoles using a temporal convolutional neural network

**DOI:** 10.3389/fnagi.2024.1437707

**Published:** 2024-07-18

**Authors:** Jae-Min Park, Chang-Won Moon, Byung Chan Lee, Eungseok Oh, Juhyun Lee, Won-Jun Jang, Kang Hee Cho, Si-Hyeon Lee

**Affiliations:** ^1^School of Electrical Engineering, Korea Advanced Institute of Science and Technology (KAIST), Daejeon, Republic of Korea; ^2^Department of Rehabilitation Medicine, Chungnam National University College of Medicine, Daejeon, Republic of Korea; ^3^Department of Biomedical Institute, Chungnam National University, Daejeon, Republic of Korea; ^4^Department of Physical Medicine and Rehabilitation, Chung-Ang University Hospital, Seoul, Republic of Korea; ^5^Department of Neurology, College of Medicine, Chungnam National University Hospital, Daejeon, Republic of Korea

**Keywords:** Parkinson's disease, freezing of gait, convolutional neural network, foot pressure, insole

## Abstract

**Backgrounds:**

Freezing of gait (FoG) is a common and debilitating symptom of Parkinson's disease (PD) that can lead to falls and reduced quality of life. Wearable sensors have been used to detect FoG, but current methods have limitations in accuracy and practicality. In this paper, we aimed to develop a deep learning model using pressure sensor data from wearable insoles to accurately detect FoG in PD patients.

**Methods:**

We recruited 14 PD patients and collected data from multiple trials of a standardized walking test using the Pedar insole system. We proposed temporal convolutional neural network (TCNN) and applied rigorous data filtering and selective participant inclusion criteria to ensure the integrity of the dataset. We mapped the sensor data to a structured matrix and normalized it for input into our TCNN. We used a train-test split to evaluate the performance of the model.

**Results:**

We found that TCNN model achieved the highest accuracy, precision, sensitivity, specificity, and F1 score for FoG detection compared to other models. The TCNN model also showed good performance in detecting FoG episodes, even in various types of sensor noise situations.

**Conclusions:**

We demonstrated the potential of using wearable pressure sensors and machine learning models for FoG detection in PD patients. The TCNN model showed promising results and could be used in future studies to develop a real-time FoG detection system to improve PD patients' safety and quality of life. Additionally, our noise impact analysis identifies critical sensor locations, suggesting potential for reducing sensor numbers.

## 1 Introduction

Parkinson's disease (PD) is a neurodegenerative disease causing motor and non-motor manifestations in older populations. Freezing of gait (FoG) is a common symptom in the PD, simply defined as “an episodic inability to generate effective stepping in the absence of any known cause other than Parkinsonism or high-level gait disorders.” It is most experienced during turning and step initiation but also when faced with spatial constraint, stress, and distraction (Giladi and Nieuwboer, 2008).

The event of FoG could affect the locomotive function and quality of life in patients with PD, even causing falls (Okuma, 2014; Perez-Lloret et al., 2014; Shah et al., 2018). The prevalence of FoG is common, measuring as 37.9% in early stages, and 64.6% for advanced stages of the disease course (Zhang et al., 2021). However, in other previous epidemiological report, the prevalence of FoG varies from 5% to 85.9% of patients (Giladi et al., 2009; Lilleeng et al., 2015). This discrepancy in observation results could be due to the ambiguity of diagnosing FoG by self-reported outcomes or observational gait analysis in a clinic. Differences between self-reported and clinically detected FoG were reported by Sawada et al., describing that 53.7% of patients who had not shown clinically detected FoG experienced self-reported FoG (Sawada et al., 2019). Therefore, FoG could be underestimated in situations where objective diagnostic tools are absent.

Not only drugs or deep brain stimulation but also non-pharmacological treatments such as auditory or visual cueing can be useful tools to alleviate FoG symptoms in PD. External cueing-augmented training can reduce the severity of FoG, improve gait parameters, and even improve upper extremity movement after training (Ginis et al., 2018). However, continuous cueing results in cueing dependency or fatigue (Spildooren et al., 2012; Ginis et al., 2017). Therefore, the demand for ‘intelligent cueing', which means providing cueing based on gait deviation or motor blocks, has been highlighted in recent times.

For the two reasons mentioned above, detecting or predicting FoG using wearable sensors has been tried by several researchers (Pardoel et al., 2019). Previous research about detecting freeze episodes in parkinsonism has used accelerometers or inertial measurement unit (IMU) sensors at various anatomical positions (Kita et al., 2017; Pepa et al., 2017; Saad et al., 2017; Prateek et al., 2018). Detecting FoG using foot pressure monitoring systems has also been used in previous reports alone (Shalin et al., 2020, 2021), or together with accelerometers (Marcante et al., 2020), or IMU data (Pardoel et al., 2020, 2021). Foot pressure monitoring systems using insoles could be a potentially useful tool in detecting FoG events in patients with PD, as they are more convenient than accelerometers or IMU sensors, which must be attached to various parts of the body. Therefore, using only a foot pressure monitoring system, there is a need to detect FoGs determined by proper algorithms. The purpose of this trial is detection of FoG using wearable plantar pressure sensors in patients with PD, and development of deep learning algorithms to detect the FoG.

## 2 Method

### 2.1 Participants

This study was prospective case-series to develop the algorithm of detecting FoG in patients with PD using plantar pressure sensor. A convenient sampling of 14 adult participants was recruited from the outpatient clinics in the neurology department of tertiary hospital. Inclusion criteria of trial was as follows; 1. Diagnosed with PD, 2. Self-reporting events of FoG at least once a week, 3. Able to perform community ambulation without a gait aid, 4. No lower limb injury or deformity that could affect the locomotive function of participants. Exclusion Criteria was as follows; 1. Presence of deep brain stimulation, 2. History of other diseases could affect the locomotive function of the participants, such as cerebral infarction, 3. Inability to understand the trial process due to cognitive dysfunction or language problem, 4. Declining to participate.

All participants visited once to collect data during the study period, and their medication dosage or schedule were not modified during the data collection process. After obtaining informed consent, basal demographic data of participants such as age, sex, height, weight, body mass index (BMI), and duration from the diagnosis of PD were also collected. The motor examination section from the Unified Parkinson's Disease Rating Scale (UPDRS III) was also assessed. Written informed consent was obtained from all participants, and the study protocol was approved by the local ethics committee of Chungnam National University Hospital (registry number: CNUH 2022-01-011).

### 2.2 Plantar pressure sensing

**Figure 1:**
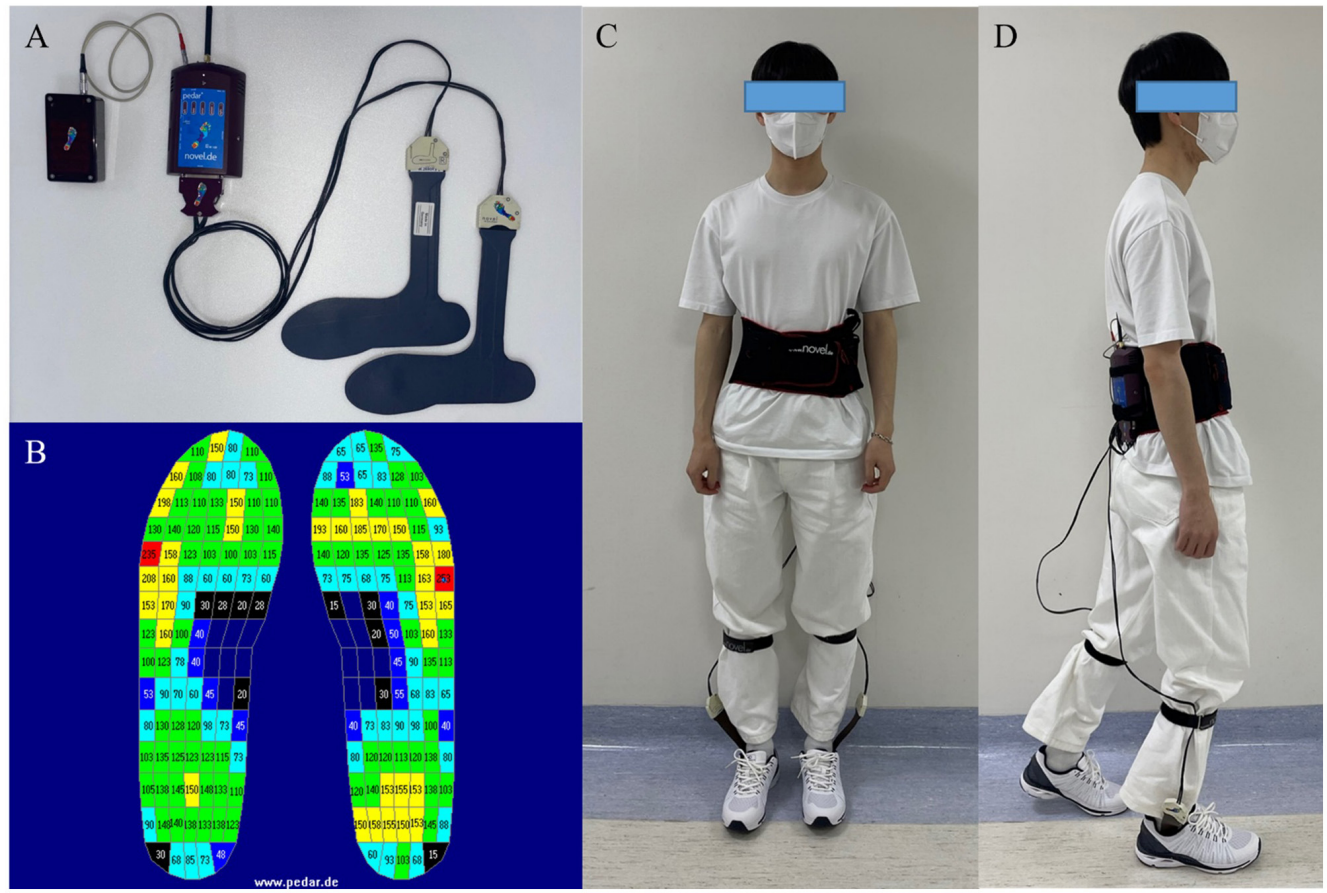
**(A)** The Pedar system (Novel GmbH, Munich, Germany). **(B)** Pedar insoles' pressure output, numeric numbers indicate the pressure measured by each sensor (kPa). **(C, D)** Example of the participant who wearing the Pedar system.

For plantar pressure analysis, the Pedar system (Novel GmbH, Munich, Germany, Figure 1) was used. The Pedar system utilizes an insole with 99 sensors to measure the pressure range from 30 to 1,200 kPa (Putti et al., 2007). It transmits data through Bluetooth telemetry and sets the data transmission rate to 50 Hz. Before each data collection, the insole was calibrated, and all the sensors were checked to ensure functionality.

There are seven insole sizes: 240–245 mm, 255–260 mm, 265–270 mm, 280–285 mm, 295–300 mm, 310–315 mm, and 320–325 mm. The subject's foot size was measured, and an appropriately sized insole was applied to ensure that the entire foot could be measured by the sensors in the insole.

### 2.3 Walking path

**Figure 2:**
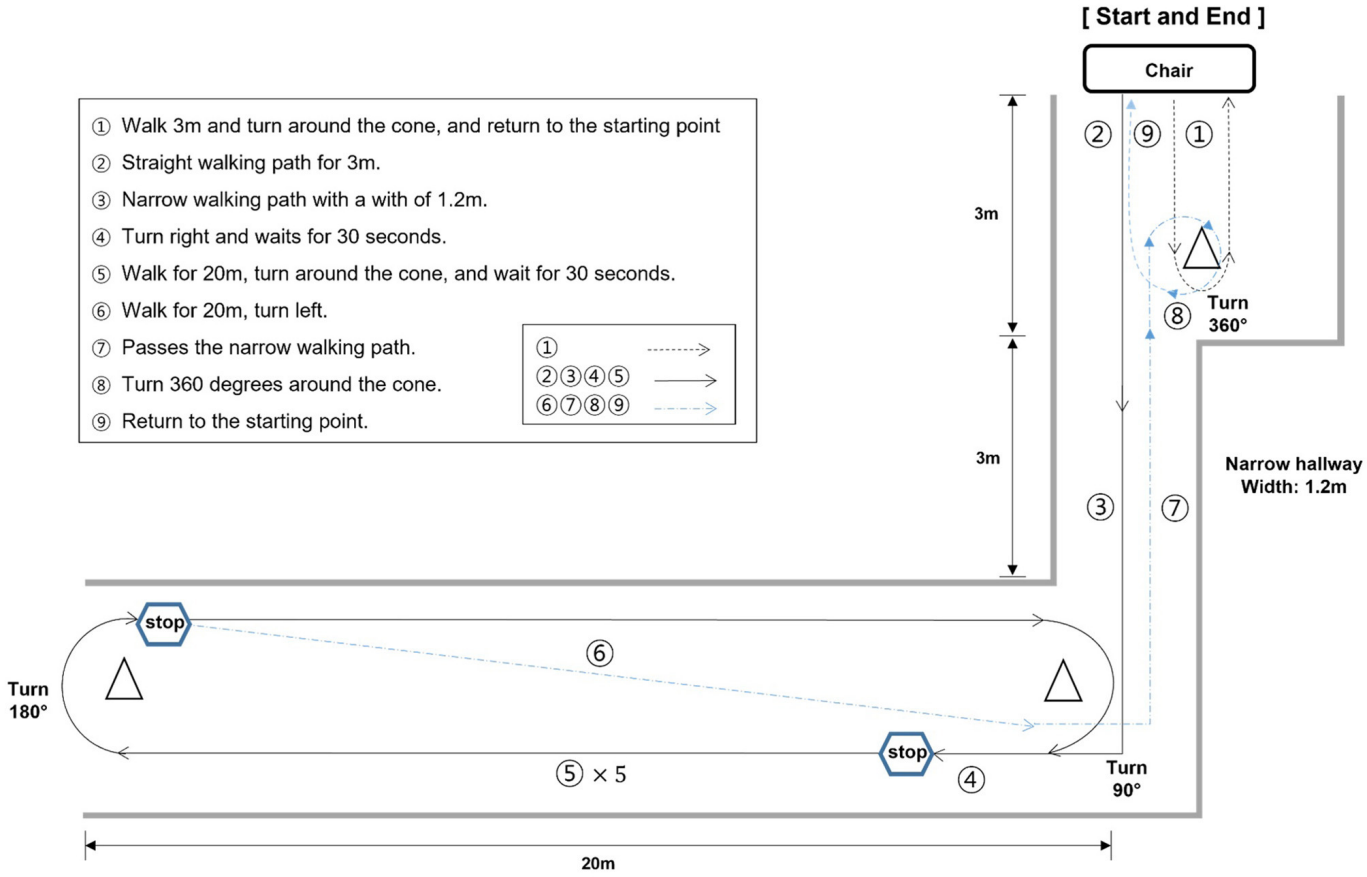
Illustration of the walking test path. The walking path was meticulously designed to incorporate scenarios where each distinct type of FoG could be observed. FoG can manifest in several ways, categorized into five types: (1) start hesitation, (2) turn hesitation, (3) apparent hesitation in tight quarters, (4) destination hesitation, and (5) open space hesitation (Fahn, 1995).

Patients were instructed to follow the walking paths (approximately 140 m). The walking path consisted of 8 tasks. The details of the tasks were as follows (Figure 2):

1. After the start sign, participants were asked to walk 3 m, turn around the cone, and return to the starting point. Then, participants waited for 30 s.
2. Walk straight for 3 m.
3. Pass through a narrow road with a width of 1.2 m for 3 m.
4. Turn right and wait for 30 seconds before entering the straight section.
5. After waiting, participants were asked to walk straight 20 m to the cone on the other side of the aisle, turn around the cone, and stop. This procedure of walking the straight aisle was repeated five times.
6. After the five repetitions, participants came back to the cone, turned left, and passed through the narrow road section again.
7. Participants were asked to turn around approximately 360 degrees at the cone before the starting point.
8. Return to the starting point.

The entire walking test was recorded using a smartphone camera to detect the clinically observed FoG events and synchronize the plantar pressure data with video clips. Each participant completed from 3 to 5 trials in test session. The first trial was a baseline evaluation, and if no FoG events were observed in the first trial, additional tasks were added to elicit FoG events. These additional tasks consisted of two parts: participants were asked to count from 100 to 0 in reverse continuously, and to hold a plastic tray with cups filled with water, walking without spilling the water. To prevent falls during the test, one or two companions supervised the entire test.

### 2.4 Detection of FoG

FoG events were visually identified using the video. Two authors, CWM and BCL, who have more than 5 years of experience in managing patients with PD thoroughly monitored the video, identified FoG events, and labeled them from the onset to termination of the FoG. During the labeling process, synchronization of the video signals and recorded data from the plantar pressure sensor was confirmed using multiple heel strike event correlations. If the occurrence of FoG events did not correspond, those events were not recorded as true events unless an agreement between the two authors was reached.

### 2.5 Data filtering

Before analysis, the data underwent a rigorous filtering process to ensure the integrity of the dataset. During the data capture phase, instances were identified where the Bluetooth telemetry suffered from transient errors, resulting in periods of inaccurate recording. To maintain the quality of our dataset, we systematically removed not only the erroneous data points but also the subsequent data that could be influenced by these errors.

Our proposed architecture, TCNN, and the baseline model, LSTM, both rely on historical data to predict future events. Particularly, the LSTM model considers up to 12.2 seconds of past data to inform its predictions. Therefore, to eliminate any potential contamination of the LSTM's input, we extended our data cleansing process to remove 12.2 seconds of data following each identified error. This precautionary step was crucial to prevent any erroneous influence on the model's performance and to ensure that the input data remained robust and reliable for both TCNN and LSTM architectures.

### 2.6 Train-test split for model evaluation

After the data filtering stage, we executed a train-test split. Participants individually allocated one trial as their testing set and the remaining trials as their training set, conducting training and testing based on this setup.

In this split, we ensured that for each participant, both the training and testing sets had at least one occurrence of a FoG event. If the training set lacked FoG events, the model would predict all data as non-FoG. Conversely, if the testing set had no FoG events, the significance of sensitivity measurement would be compromised. This careful allocation of trials aimed to provide a balanced representation of FoG episodes in both sets. Our criteria mandated the presence of at least two trials with FoG episodes after data filtering, which was a determinant factor in participant selection.

### 2.7 Mapping sensor data to a structured matrix

To prepare the input for our neural network, we transformed the sensor data into a structured matrix form that corresponds to the anatomical layout of a foot. As described in Section 2.2, the pedar system consists of 99 sensors on each foot, totaling 198 pressure sensors when both feet are considered. We organized the vector composed of the output from these 198 pressure sensors into a 15 × 14 matrix. Considering that 15 multiplied by 14 equals 210, zeros were strategically inserted to fill the gaps in areas without sensors. This arrangement creates a visual and spatial representation resembling a footprint. The resulting matrix is illustrated in Supplementary Figure S5.

### 2.8 Normalization of sensor data

To normalize the sensor data across all trials, a normalization procedure was applied to the collected sensor values. This process was designed to scale the data such that, at the moment of maximum pressure recorded by the sensors on each foot, the sum of all sensor values would equal to 1,000.

### 2.9 Baseline models

For comparative purposes, we considered two baseline models: a Convolutional Neural Network (CNN) (Shalin et al., 2020) and a Long Short-Term Memory (LSTM) (Shalin et al., 2021) model. Detailed descriptions of these baseline models, including their architectures and implementation details, are provided in Section 3 of the Supplementary material.

### 2.10 Temporal convolutional neural network (TCNN)

In this study, we propose a TCNN, designed to handle multi-temporal data. TCNN differs from the baseline CNN by taking input from multiple time frames of insole matrices. The input to TCNN is structured as a tensor of shape *t*×15 × 14, where each temporal slice has the form of 15 × 14 matrix explained in Section 2.7, representing sensor data at a specific time instance.

To deal with this input structure, we adapted the ResNet-18 architecture to process the temporal dimension *t* as its input channel. This modification allows the TCNN to analyze not just spatial but also temporal patterns across different moments in the gait cycle, enhancing its ability to detect subtle variations and dynamics indicative of FoG episodes.

The TCNN in our study was designed to capture both fine and coarse temporal changes relevant to gait analysis, particularly for detecting FoG episodes. To achieve this, we employed a dual-scale approach in feeding the input to the TCNN. The model concurrently examine two distinct temporal scales: one scale targets finer details by examining 18 frames with a 4-frame interval, while the other focuses on broader, long-term changes by considering 6 frames at a 13-frame interval. The vectors from both the fine and coarse scales are then concatenated to form a unified input, which is fed into the model. This concatenation allows the TCNN to analyze both detailed short-term dynamics and more extended temporal patterns.

### 2.11 Data augmentation techniques

To bolster the robustness of our TCNN and baseline models, a series of probabilistic data augmentation techniques were employed. These techniques not only introduce variability to simulate different gait conditions but also help the models adapt to diverse scenarios:

- **Temporal scaling:** In the TCNN, the timing of input frames was varied within a range of ±40%, and for the LSTM, within ±50%. This scaling was done to mimic natural gait speed fluctuations, effectively varying the speed of the sequence between 60% (50%) to 140% (150%) of its original rate.
- **Horizontal flip:** A horizontal flip was applied to the input data to represent a mirrored version of the gait pattern. With a probability of 30%, we flipped all frames in an input sequence uniformly for both TCNN and CNN, ensuring that either the entire sequence was flipped or none of it was, avoiding partial flips.
- **Global scaling:** To account for sensor sensitivity, we uniformly adjusted the magnitude of all sample values within each input. This scaling was applied globally, ranging from 0.1 to 1.9 times the original value for each input sequence.
- **Gaussian blur:** To simulate sensor imprecision and minor transient changes in pressure distribution, we applied a Gaussian blur with a 50% probability. We used a kernel size of 3 and a standard deviation uniformly selected between 0.1 and 2, applying this blur consistently across all frames in an input sequence uniformly for both TCNN and CNN. This technique adds a realistic touch of uncertainty to the input data, akin to natural sensor behavior.

### 2.12 Validation of developed models

We evaluated the performances of LSTM, CNN, and the proposed TCNN models using various performance metrics. Considering not only accuracy but also the imbalance between FoG and non-FoG data, we measured precision, sensitivity, specificity and F1 score (Hicks et al., 2022). Each metric was calculated by summing the true positives, true negatives, false positives, and false negatives across all participants and over three repeated experiments with different random seeds. The reported values represent the performance metrics derived from these aggregated sums.

### 2.13 Impact of sensor noise analysis

Due to the challenges and costs associated with maintaining numerous sensors, it is crucial to reduce their number to simplify the device and decrease expenses. To determine which sensors are essential, we systematically introduced noise to various sensors and assessed the impact on performance. The specific selection of the three sensor locations–big toe, forefoot, and heel–was influenced by their frequent usage in existing smart insole systems (Sazonov et al., 2011). This approach allows us to identify which sensors are critical for maintaining performance integrity and should be prioritized in streamlined device designs.

- Raw: No noisy sensors
- Big toe: 4 noisy sensors near the big toe
- Forefoot: 4 noisy sensors at the forefoot
- Heel: 4 noisy sensors at the heel

For each of the above three types, we considered a constant output value of noisy sensors, which is 50 per sensor, resulting in a total noise of 200 per foot as each foot has 4 noisy sensors. The exact positions of the noise are illustrated in Supplementary Figures S6–S8.

## 3 Results

From the original cohort of 14, only four participants' data satisfied the criteria of Section 2.6. The basal demographics and clinical characteristics of selected four participants were described in Section 1 of Supplementary material. Detailed data amounts of FoG and non-FoG in each and total trials was depicted in the Supplementary Figures S1–S4.

We trained LSTM, CNN, and TCNN models, and evaluated the performance of each model on the test set. We note that the models were trained solely on raw data without artificially added noise, and the three types of noise described in Section 2.12 are applied to the test set (Supplementary Figures S5, S6).

The experimental results can be found in Table 1.

**Table 1:** Comparison of performance metrics across different models and noisy sensor positions for the FoG detection task.

	**Model**	**Raw**	**Big toe**	**Forefoot**	**Heel**
Accuracy	LSTM	0.69	0.63	0.63	0.69
		CNN	0.98	0.89	**0.77**	0.93
		TCNN	**0.99**	**0.90**	0.58	**0.97**
Precision	LSTM	0.04	0.03	0.03	0.03
		CNN	0.36	0.08	0.03	0.01
		TCNN	**0.68**	**0.08**	**0.03**	**0.10**
Sensitivity	LSTM	0.74	**0.76**	0.76	**0.66**
		CNN	0.70	0.54	0.47	0.02
		TCNN	**0.88**	0.49	**0.93**	0.14
Specificity	LSTM	0.69	0.63	0.63	0.69
		CNN	0.98	0.89	**0.78**	0.94
		TCNN	**0.99**	**0.91**	0.57	**0.98**
F1 Score	LSTM	0.07	0.06	0.06	0.06
		CNN	0.48	0.13	0.06	0.01
		TCNN	**0.76**	**0.14**	**0.07**	**0.12**
TP	LSTM	2,195	2,262	2,260	1,963
		CNN	2,099	1,606	1,401	72
		TCNN	2,623	1,454	2,771	423
FP	LSTM	56,746	67,834	67,426	55,655
		CNN	3,693	19,477	40,046	10,875
		TCNN	1,253	16,868	78,126	3,741
TN	LSTM	125,066	113,978	114,386	126,157
		CNN	178,119	162,335	141,766	170,937
		TCNN	180,559	164,944	103,686	178,071
FN	LSTM	790	723	725	1,022
		CNN	886	1,379	1,584	2,913
		TCNN	362	1,531	214	2,562

FoG, Freezing of Gait; LSTM, Long Short-Term Memory; CNN, Convolutional Neural Network; TCNN, Temporal Convolutional Neural Network; TP, True Positives; FP, False Positives; TN, True Negatives; FN, False Negatives.

FoG and non-FoG episodes are considered positive and negative, respectively. Bold values indicate the best performance for each metric and sensor position.

In most situations and metrics, our TCNN model demonstrated superior performance compared to both LSTM and CNN models. In raw situations without noise, our TCNN achieved an accuracy of 0.99, precision of 0.68, sensitivity of 0.88, specificity of 0.99, and an F1 score of 0.76 in detecting FoGs, consistently outperforming LSTM and CNN. In comparison, the LSTM model achieved an F1 score of 0.07, and the CNN model achieved an F1 score of 0.48 in detection of FoG. The detailed results of LSTM, CNN, and TCNN for each participant can be found in the Supplementary Tables S2–S5.

The analysis presented in Table 2 reveals distinct impacts of various noise situation on the performance of the TCNN depending on each participant's CoP distribution and sensor placement. For instance, participants ID1, ID3, and ID4, who predominantly exhibit CoP concentrated at forefoot when FoG occured, were exhibited decreased specificity due to big toe and forefoot noise, which would likely misclassify non-FoG as FoG. Conversely, heel noise significantly reduced sensitivity for ID1, ID3, and ID4. This effect is likely due to the heel noise shifting the CoP backward, causing FoG events to be misclassified as non-FoG. This results in an increase in false negatives and, consequently, a decrease in sensitivity, aligning with our predictions.

**Table 2:** CoP scatter plot and performance of TCNN for individual test datasets.

	**ID: 1**		**ID: 2**		**ID: 3**		**ID: 4**	
	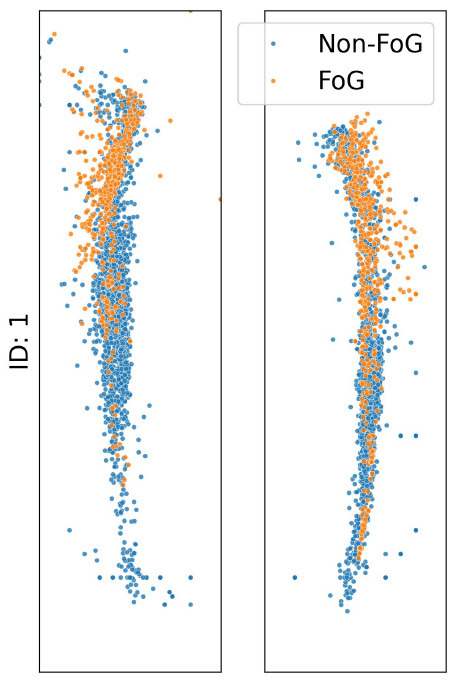		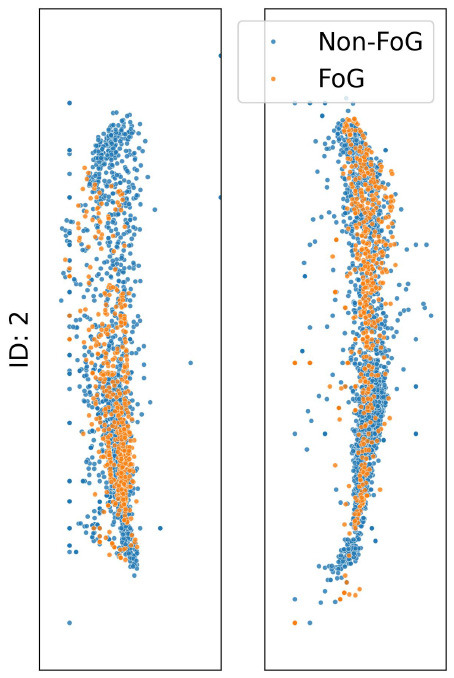		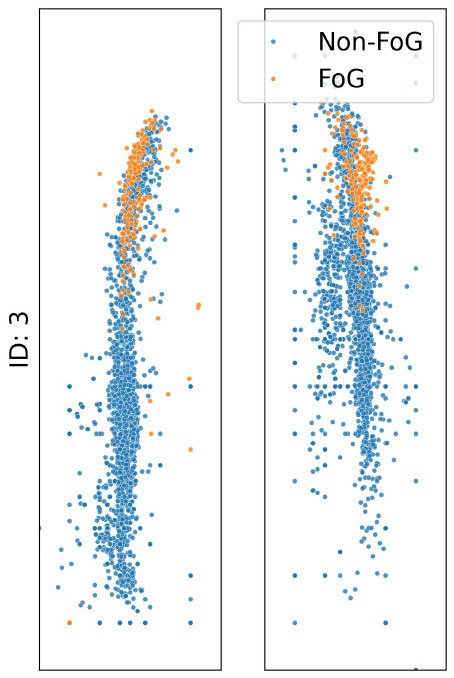		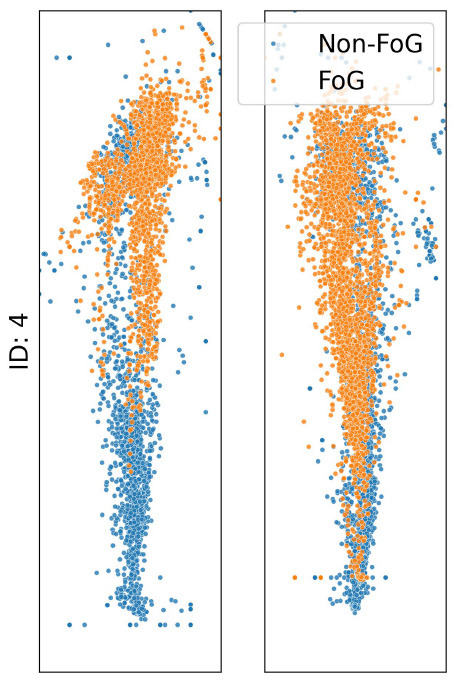	
**16.5-1,15.5498pt Setup**	**Sensitivity**	**Specificity**	**Sensitivity**	**Specificity**	**Sensitivity**	**Specificity**	**Sensitivity**	**Specificity**
Raw	0.81	0.99	0.68	0.99	1.00	1.00	0.96	1.00
Big toe	0.93	0.83	0.39	0.86	0.90	1.00	0.44	1.00
Forefoot	0.24	1.00	1.00	0.00	0.84	0.82	1.00	0.00
Heel	0.04	1.00	0.57	0.90	0.00	1.00	0.00	1.00

FoG, Freezing of Gait; CoP, Center of Pressure; TCNN, Temporal Convolutional Neural Network.

FoG and non-FoG episodes are considered positive and negative, respectively.

The case of ID2 presented a more complex scenario. With the CoP distributed both at the big toe, forefoot and heel during FoG, it was anticipated that big toe and forefoot noise would reduce both sensitivity and specificity by misclassifying FoG events as non-FoG. However, heel noise maintained high specificity, deviating from predictions.

## 4 Discussion

### 4.1 Summary of findings

Our examination highlights the TCNN model's capability to discern FoG events compared to other models (CNN, LSTM) in various test scenarios. The noise introduced in the three sensor locations (big toe, forefoot, and heel area) caused the performance of the algorithms to decrease compared to the raw scenarios. We also observed fluctuations in performance among the participants, which could be attributed to the varying amounts of FoG data.

### 4.2 Comparison with previous studies

In our experiments TCNN model achieved a sensitivity of 0.88 and a specificity of 0.99 using foot pressure sensor data, outperforming the other models, with the CNN achieving 0.70 and 0.98, and the LSTM achieving 0.74 and 0.69. Compared to other studies, our results are competitive. In comparison, Shalin et al. (2020) reported 0.92 and 0.96 with a CNN, and Shalin et al. (2021) reported 0.82 and 0.89 with a two-layer LSTM model. Additionally, Pardoel et al. (2022) showed that using foot pressure data from only the side more severely affected by PD, still yielded reasonable performance with a sensitivity of 0.74 and a specificity of 0.85. Studies using IMU data showed varied performance, such as San-Segundo et al. (2019) achieving 0.95 and 0.75 with a CNN, and Borzì et al. (2021) reporting 0.94 and 0.92 for those on dopaminergic therapy, and 0.94 and 0.85 for those not on therapy using decision trees and support vector machine models. Combining IMU and foot pressure sensor data is also being researched, with Marcante et al. (2020) achieving 0.96 and 0.94 using a rule-based algorithm. Tǎutan et al. (2020) reporting 0.93 and 0.87 with a CNN, and Pardoel et al. (2021) achieving 0.93 and 0.86 with boosted decision trees.

### 4.3 Practical implications

In this study, we addressed the need for objective and reliable methods for detecting FoGs. Current FoG detection methods rely on subjective assessments, such as questionnaires and video observations, which could be inconsistent and prone to bias. To overcome these limitations, we proposed a novel approach that combines foot pressure sensors with a TCNN model. Our TCNN model enhances the precision and reliability of FoG detection by analyzing both spatial and temporal patterns in sensor data, providing significant advantages over traditional methods.

By employing sensor-based detection, our TCNN model could facilitate more accurate evaluations of drug or other clinical therapy efficacy, surpassing the accuracy of subjective measures. This objectivity is crucial for better understanding the impacts of therapeutic interventions on FoG episodes. Furthermore, automated FoG detection reduces the necessity for labor-intensive video reviews.

In addition, the TCNN model supports adaptive cueing systems that activate only when a FoG episode is detected, thus reducing patient fatigue associated with continuous cues. This adaptive approach could enhance patient comfort and treatment efficacy. Integrating the TCNN model with wearable devices allows for personalized and timely interventions, improving the management of FoG. Such real-time adaptability is essential for responding effectively to the dynamic nature of FoG episodes.

### 4.4 Limitations and future directions

Significantly, performance disparities among participants were evident, with those experiencing more frequent FoG events exhibiting better F1 scores. This observation is aligned with prior researches (Shalin et al., 2020, 2021), suggesting that the volume of positive data can crucially impact model accuracy. As shown in Nguyen (2019) and DeVries et al. (2021), F1 scores can significantly decrease in imbalanced datasets due to low precision, which typically occurs when the proportion of positive samples in the test set is low. Future studies would benefit from utilizing datasets with more extensive FoG data to further refine the algorithms.

We directly introduced various noise situations into the study and observed significant performance impacts at the big toe, forefoot, and heel sensor locations. Our approach differs from previous methods (Pardoel et al., 2020, 2022) that addressed sensor noise for by thresholding feature values but did not involve direct noise introduction, thereby making a novel contribution to sensor-based FoG detection research. Our findings will be useful when trying to reduce the number of sensors within each foot, as we identified the big toe, forefoot, and heel sensors as critical.

A limitation of this study was the restricted number of participants. Despite involving 14 participants in multiple trials, only four remained after applying the participant selection criteria mentioned in Section 2.6. This was due to the limited occurrence of FoG across multiple trials for some participants. To overcome these challenges, future studies could aim to include larger datasets with a higher incidence of FoG events, which would provide more robust data for training and evaluating our models. Our experiments demonstrated the critical importance of sensors located at the big toe, forefoot, and heel. These findings suggest the potential for reducing the number of sensors by focusing on key areas like the big toe, forefoot, and heel. This reduction could lower costs, simplify device setups, and enhance user comfort, making the technology more practical for everyday clinical use. Future research should explore optimizing sensor placement to maintain accuracy while minimizing device complexity, thereby validating our findings and assessing the scalability of the sensor system in a wider range of clinical settings.

## Data availability statement

The raw data supporting the conclusions of this article will be made available by the authors, without undue reservation.

## Ethics statement

The studies involving humans were approved by the Local Ethics Committee of Chungnam National University Hospital (registry number: CNUH 2022-01-011). The studies were conducted in accordance with the local legislation and institutional requirements. The participants provided their written informed consent to participate in this study.

## Author contributions

J-MP: Data curation, Formal analysis, Methodology, Software, Validation, Visualization, Writing – original draft, Writing – review & editing. C-WM: Conceptualization, Funding acquisition, Investigation, Methodology, Writing – original draft, Writing – review & editing. BL: Investigation, Methodology, Supervision, Writing – original draft, Conceptualization. EO: Investigation, Writing – review & editing. JL: Investigation, Methodology, Writing – original draft. W-JJ: Formal analysis, Methodology, Validation, Writing – review & editing. KC: Conceptualization, Writing – review & editing, Funding acquisition, Formal analysis, Methodology, Validation. S-HL: Formal analysis, Funding acquisition, Methodology, Resources, Supervision, Writing – review & editing.
