# Supplementary material for: Detection of freezing of gait in Parkinson's disease from foot-pressure sensing insoles using a temporal convolutional neural network
Source: Front Aging Neurosci. 2024 Jul 18;16:1437707. doi: 10.3389/fnagi.2024.1437707 (PMC11291202; doi:10.3389/fnagi.2024.1437707)
Supplement: Supplementary file 1 [file Data_Sheet_1.pdf]

Supplementary Material

1 INFORMATION ABOUT THE PARTICIPANTS

Table S1 provides detailed information about the participants involved in the experiment.

Table S1. Summary of Participant Data.

ID	Age	Sex	Height	Weight	BMI	Disease duration (yrs)	UPDRS III	Number of tested trial	Trial total time	Number of FOG episodes	FOG total time (s)
1	77	M	162.3	65.9	25.0	15	26	4	2307	8	12.04
2	72	F	148.5	57.2	25.9	4	18	4	1635	7	11.60
3	69	M	161.0	68.0	26.2	7	36	4	1713	4	4.50
4	62	M	169.2	62.7	21.9	20	29	4	1835	6	58.80

Figures S1- S4 represent the number of data points for each participant's trials. These include the count of non-FoG data, FoG data, total data per trial, and the count of FoG and non-FoG data across all trials.

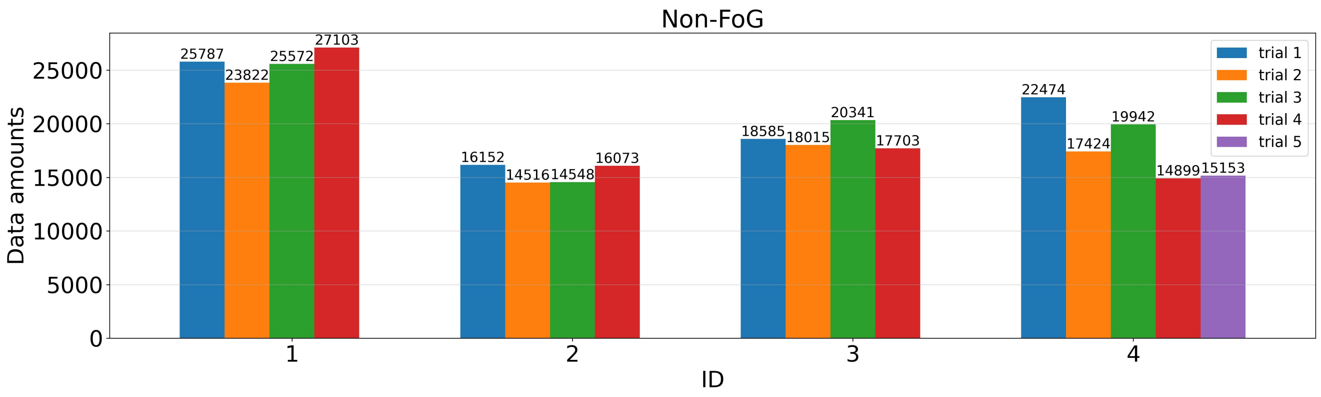

Figure S1: Number of non-FoG data points for each trial.

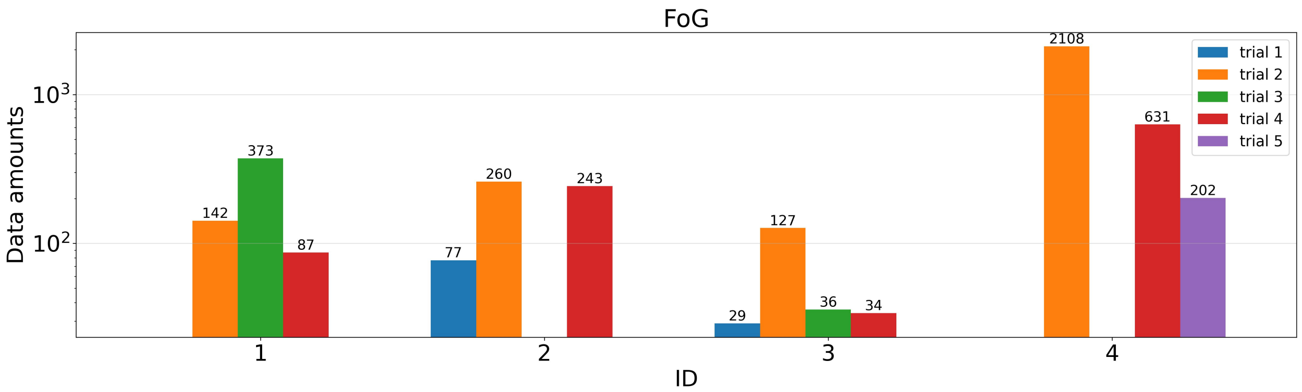

Figure S2: Number of FoG data points for each trial.

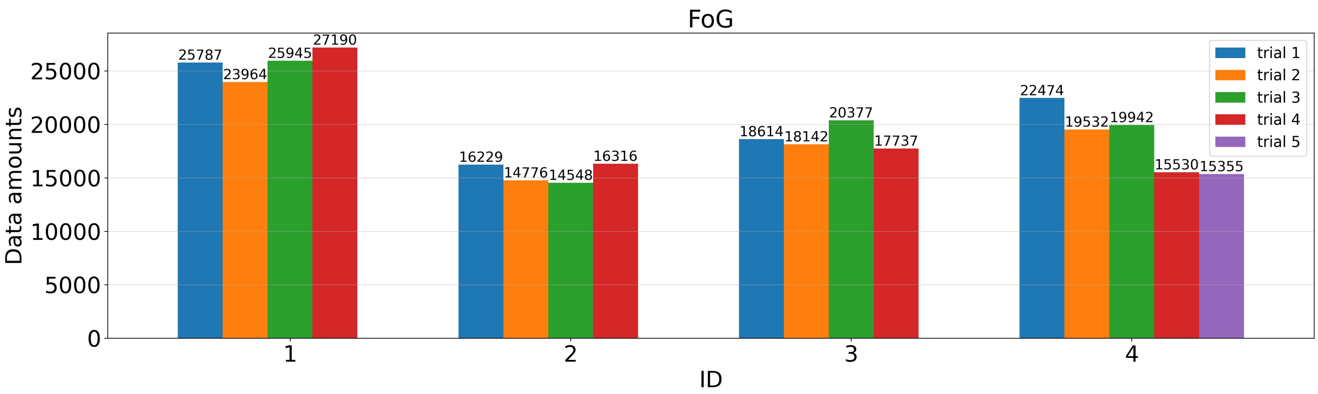

Figure S3: Number of total data points for each trial.

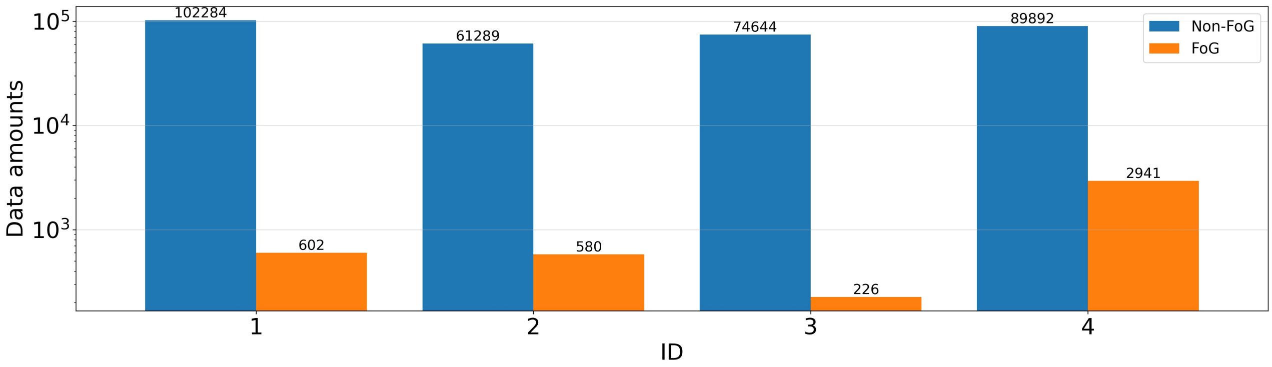

Figure S4: Total number of non-FoG and FoG data points for each ID.

2 DATA MAPPING AND NOISE OF THE PEDAR INSOLE

Figure S5 illustrates how left and right pedar sensors with numbers 1 to 99 are mapped into a 15×14 matrix. The numbers correspond to the sensor numbers in the original data vector. Figures S6, S7, and S8 depict locations of sensors with constant noise for each noise situation.

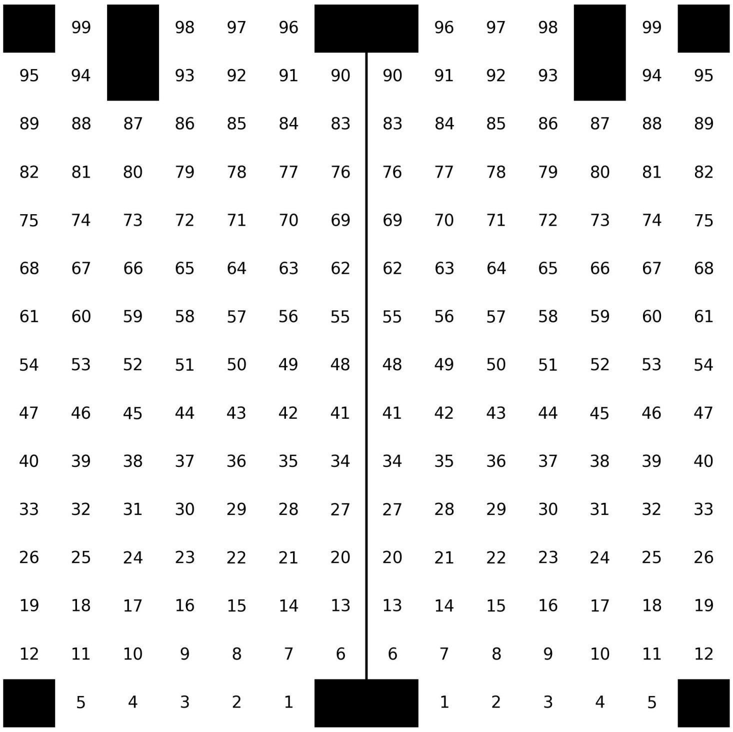

Figure S5: The mapping of sensor data to the matrix form. The sensors from 1 to 99 on the left foot are mapped to the left side of the figure (1 to 99), and the sensors from 1 to 99 on the right foot are mapped to the right side of the figure (1 to 99).

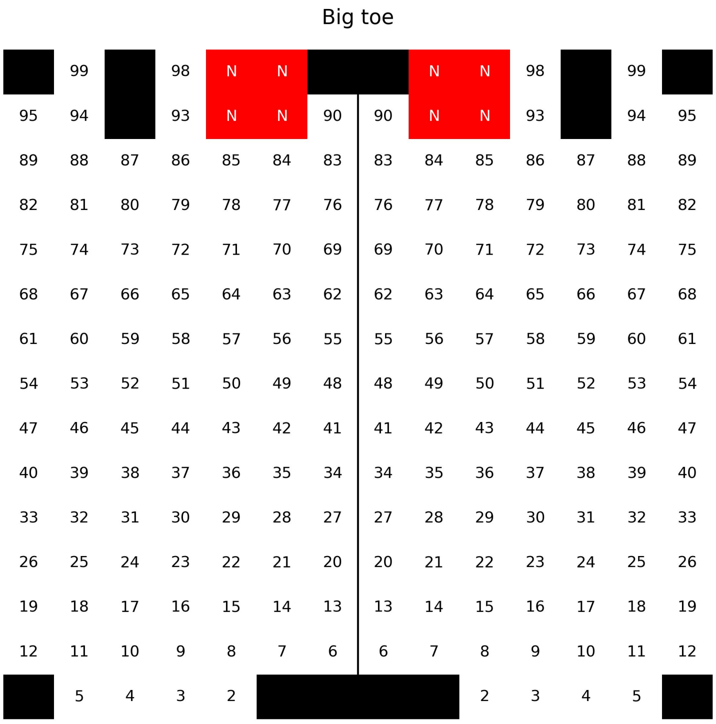

Figure S6: This shows the big toe noise situation. The red areas indicate noisy sensors, each with a constant value of 50.

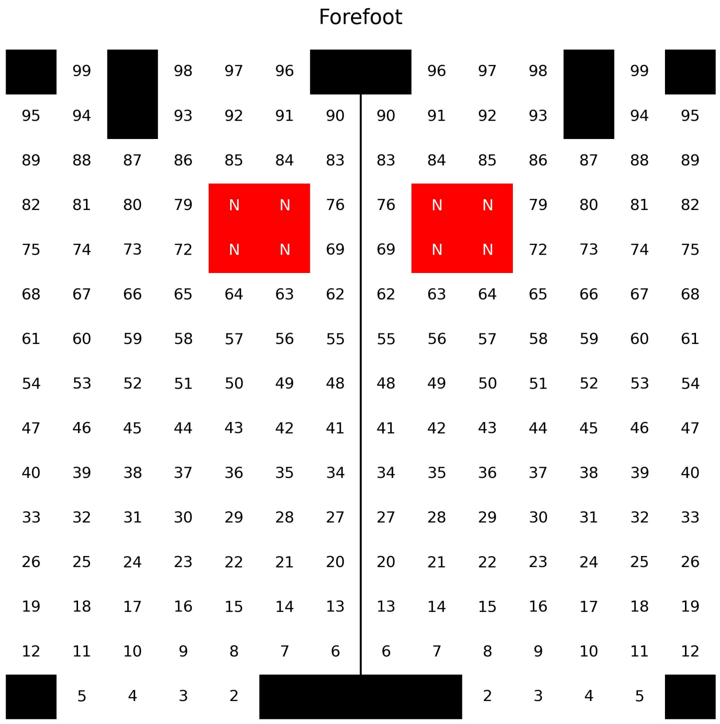

Figure S7: This shows the forefoot noise situation. The red areas indicate noisy sensors, each with a constant value of 50.

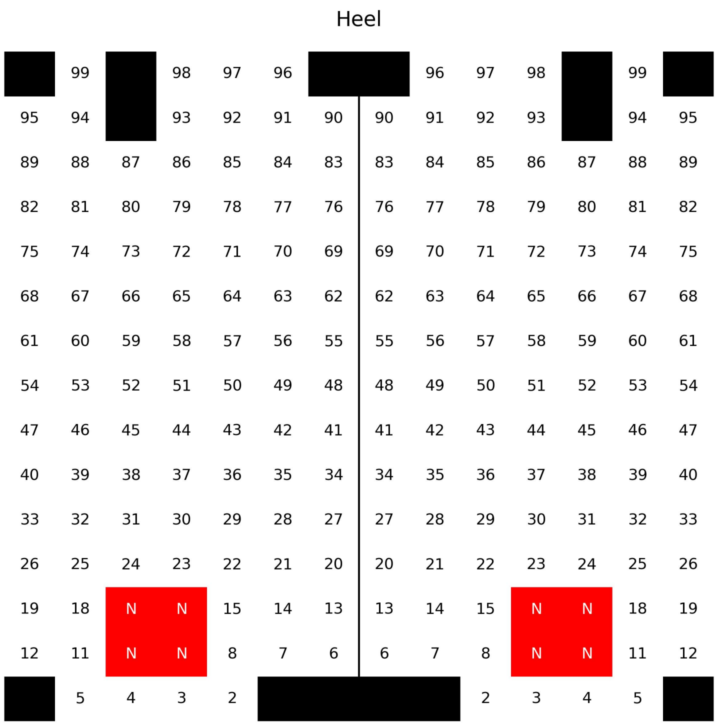

Figure S8: This shows the heel noise situation. The red areas indicate noisy sensors, each with a constant value of 50.

3 BASELINE MODELS

3.1 CNN

The CNN model is based on the ResNet-18 (He et al., 2016) architecture. This model takes the instantaneous sensor data, represented by a 15×14 matrix, as input, and determines whether the participant is currently experiencing FoG event or not. While the original ResNet-18 architecture typically handles 3-channel input (e.g., RGB images), we modified the input channel size to 1 to accommodate the sensor data. This adaptation allowed us to treat each pressure matrix as a single-channel input.

3.2 LSTM

In our study, the input of the LSTM model was designed with 24 dimensions, based on predefined criteria. These dimensions are composed of multiple features: Center of Pressure (CoP) coordinates in four dimensions, Ground Reaction Force (GRF) values in two dimensions, and the fraction of total GRF in two dimensions, each considered along with their first and second temporal derivatives. This results in 24 dimensions, calculated as follows: CoP with 4 dimensions, GRF with 2 dimensions, and the fraction of GRF with 2 dimensions, each multiplied by 3 to account for the original values and their first and second temporal derivatives (i.e., $(4 + 2 + 2) \times 3 = 24$).

- **CoP Coordinates:** We calculated the CoP coordinates for both the medial-lateral (x-axis) and anterior-posterior (y-axis) directions. These coordinates show how pressure is distributed across the foot. For example, if the CoP is located centrally, it indicates an even pressure distribution across the foot, which is crucial for analyzing balance and weight shift during gait.

- **GRF:** The GRF for each foot was determined by summing the forces applied to all sensors. This total force represents the interaction between the foot and the ground, providing insight into the dynamic forces during walking.
- **Fraction of Total GRF:** The ratio of the GRF of each foot to the combined GRF of both feet. This ratio illustrates how each foot's GRF compares to the total GRF of both feet combined, useful for understanding how body weight is distributed between the two feet during gait.
- **Temporal Derivatives:** The first and second temporal derivatives of the CoP coordinates, GRF values, and the fraction of total GRF values were computed to capture the rate of change and acceleration of pressure distribution, respectively. The first derivative indicates how fast the pressure is changing over time, while the second derivative measures the acceleration of this change.

The LSTM model in our study was specifically adapted to our dataset through the optimization process described in Sec. 4. We established an architecture with two hidden layers, with each layer comprising 33 nodes. The model was also configured with dropout (Srivastava et al., 2014) to prevent overfitting, and bidirectional LSTM layers (Graves and Schmidhuber, 2005) to enhance its ability to learn dependencies in both forward and backward directions.

4 HYPERPARAMETER OPTIMIZATION

To ensure the optimal performance of our baseline models and TCNN, we employed a Bayesian optimization approach for hyperparameter tuning. We utilized Weights & Biases (wandb) sweeps to automate and efficiently conduct this search (Biewald, 2020). The following details provide an overview of the hyperparameters and their corresponding values for each of the baseline models after optimization:

4.1 Temporal Convolutional Neural Network (TCNN)

- **Batch Size**: 32
- **Optimizer**: AdamW
  - Learning Rate: 0.0001423409219001875
  - Weight Decay: 0.003674121261172089
- **Scheduler**: Cosine Annealing
  - Gamma: 0.6461231986707445
  - Initial period: 29
  - Multiplicative factor: 2
  - Maximum learning rate: 0.11025804577175805
  - Number of epochs for warmup: 9

4.2 Long Short-Term Memory (LSTM)

- **Batch Size**: 32
- **Optimizer**: AdamW
  - Learning Rate: 0.0001423409219001875
  - Weight Decay: 0.003674121261172089
- **Scheduler**: Cosine Annealing
  - Gamma: 0.6461231986707445

- Initial period: 29
- Multiplicative factor: 2
- Maximum learning rate: 0.11025804577175805
- Number of epochs for warmup: 9

4.3 Convolutional Neural Network (CNN)

In our experiments, we used the CNN model as a special case of the TCNN, where the temporal window was restricted to size 1. Given this configuration, we applied the same optimized hyperparameters from the TCNN model to the CNN. This approach proved effective, as the CNN was trained well with these parameters. Therefore, the hyperparameters for the CNN are identical to those used in the TCNN.

5 INDIVIDUAL PERFORMANCE METRICS FOR EACH PARTICIPANT

Tables S2, S3, S4, and S5 present the algorithm performance for each of the four participants. The performance metrics include accuracy, precision, sensitivity, specificity, and F1 score.

REFERENCES

Biewald, L. (2020). Experiment tracking with weights and biases Software available from wandb.com

Graves, A. and Schmidhuber, J. (2005). Framewise phoneme classification with bidirectional lstm and other neural network architectures. *Neural networks* 18, 602–610

He, K., Zhang, X., Ren, S., and Sun, J. (2016). Deep residual learning for image recognition. In *Proceedings of the IEEE conference on computer vision and pattern recognition*. 770–778

Srivastava, N., Hinton, G., Krizhevsky, A., Sutskever, I., and Salakhutdinov, R. (2014). Dropout: a simple way to prevent neural networks from overfitting. *The journal of machine learning research* 15, 1929–1958

Table S2. Performance of our TCNN and the baselines (LSTM, CNN) for ID1.

	Model	Normal	Bigtoe	Forefoot	Heel
Accuracy	LSTM	0.64	0.60	0.60	0.60
	CNN	0.99	0.99	0.81	1.00
	TCNN	0.99	0.84	0.99	1.00
Precision	LSTM	0.01	0.01	0.01	0.01
	CNN	0.17	0.03	0.01	-
	TCNN	0.21	0.02	0.25	0.65
Sensitivity	LSTM	0.65	0.67	0.67	0.67
	CNN	0.79	0.03	0.67	0.00
	TCNN	0.81	0.93	0.24	0.04
Specificity	LSTM	0.64	0.60	0.60	0.60
	CNN	0.99	1.00	0.81	1.00
	TCNN	0.99	0.83	1.00	1.00
F1 Score	LSTM	0.01	0.01	0.01	0.01
	CNN	0.28	0.03	0.03	-
	TCNN	0.34	0.04	0.25	0.08
TP	LSTM	169	174	174	174
	CNN	205	8	174	0
	TCNN	212	242	63	11
FP	LSTM	24,569	27,619	27,592	27,558
	CNN	980	312	13,016	0
	TCNN	782	11,402	188	6
TN	LSTM	44,512	41,462	41,489	41,523
	CNN	68,101	68,769	56,065	69,081
	TCNN	68,299	57,679	68,893	69,075
FN	LSTM	92	87	87	87
	CNN	56	253	87	261
	TCNN	49	19	198	250

Table S3. Performance of our TCNN and the baselines (LSTM, CNN) for ID2.

	Model	Normal	Bigtoe	Forefoot	Heel
Accuracy	LSTM	0.36	0.90	0.90	0.90
	CNN	0.97	0.98	0.46	0.97
	TCNN	0.99	0.85	0.02	0.89
Precision	LSTM	0.03	0.03	0.03	0.03
	CNN	0.30	-	0.03	0.02
	TCNN	0.63	0.05	0.02	0.10
Sensitivity	LSTM	0.95	0.15	0.15	0.15
	CNN	0.57	0.00	0.81	0.01
	TCNN	0.68	0.39	1.00	0.57
Specificity	LSTM	0.34	0.91	0.91	0.91
	CNN	0.97	1.00	0.46	0.99
	TCNN	0.99	0.86	0.00	0.90
F1 Score	LSTM	0.05	0.05	0.05	0.05
	CNN	0.39	-	0.05	0.01
	TCNN	0.65	0.09	0.04	0.17
TP	LSTM	693	109	108	109
	CNN	415	0	590	5
	TCNN	493	284	729	412
FP	LSTM	24,760	3,352	3,317	3,341
	CNN	978	0	20,558	289
	TCNN	286	5,361	37,712	3,735
TN	LSTM	13,013	34,421	34,456	34,432
	CNN	36,795	37,773	17,215	37,484
	TCNN	37,487	32,412	61	34,038
FN	LSTM	36	620	621	620
	CNN	314	729	139	724
	TCNN	236	445	0	317

Table S4. Performance of our TCNN and the baselines (LSTM, CNN) for ID3.

	Model	Normal	Bigtoe	Forefoot	Heel
Accuracy	LSTM	0.83	0.72	0.72	0.72
	CNN	0.98	0.92	0.99	0.75
	TCNN	1.00	1.00	0.82	1.00
Precision	LSTM	0.01	0.01	0.01	0.01
	CNN	0.12	0.02	0.09	0.01
	TCNN	0.55	0.47	0.01	-
Sensitivity	LSTM	1.00	1.00	1.00	1.00
	CNN	1.00	0.62	0.35	0.66
	TCNN	1.00	0.90	0.84	0.00
Specificity	LSTM	0.83	0.72	0.72	0.72
	CNN	0.98	0.92	0.99	0.75
	TCNN	1.00	1.00	0.82	1.00
F1 Score	LSTM	0.03	0.02	0.02	0.02
	CNN	0.21	0.04	0.14	0.01
	TCNN	0.71	0.62	0.02	-
TP	LSTM	102	102	102	102
	CNN	102	63	36	67
	TCNN	102	92	86	0
FP	LSTM	7,058	11,684	11,701	11,668
	CNN	760	3,239	370	10,582
	TCNN	82	105	7,402	0
TN	LSTM	35,074	30,448	30,431	30,464
	CNN	41,372	38,893	41,762	31,550
	TCNN	42,050	42,027	34,730	42,132
FN	LSTM	0	0	0	0
	CNN	0	39	66	35
	TCNN	0	10	16	102

Table S5. Performance of our TCNN and the baselines (LSTM, CNN) for ID4.

	Model	Normal	Bigtoe	Forefoot	Heel
Accuracy	LSTM	0.97	0.27	0.28	0.61
	CNN	0.96	0.53	0.79	0.95
	TCNN	0.99	0.97	0.05	0.95
Precision	LSTM	0.77	0.07	0.07	0.11
	CNN	0.59	0.09	0.09	0.00
	TCNN	0.95	1.00	0.05	-
Sensitivity	LSTM	0.65	0.99	0.99	0.83
	CNN	0.73	0.81	0.32	0.00
	TCNN	0.96	0.44	1.00	0.00
Specificity	LSTM	0.99	0.23	0.24	0.60
	CNN	0.97	0.51	0.81	1.00
	TCNN	1.00	1.00	0.00	1.00
F1 Score	LSTM	0.71	0.13	0.13	0.19
	CNN	0.65	0.16	0.14	-
	TCNN	0.95	0.61	0.10	-
TP	LSTM	1,231	1,877	1,876	1,578
	CNN	1,377	1,535	601	0
	TCNN	1,816	836	1,893	0
FP	LSTM	359	25,179	24,816	13,088
	CNN	975	15,926	6,102	4
	TCNN	103	0	32,824	0
TN	LSTM	32,467	7,647	8,010	19,738
	CNN	31,851	16,900	26,724	32,822
	TCNN	32,723	32,826	2	32,826
FN	LSTM	662	16	17	315
	CNN	516	358	1,292	1,893
	TCNN	77	1,057	0	1,893
